# Hospitalised patients with breakthrough COVID-19 following vaccination during two distinct waves in Israel, January to August 2021: a multicentre comparative cohort study

**DOI:** 10.2807/1560-7917.ES.2022.27.20.2101026

**Published:** 2022-05-19

**Authors:** Tal Brosh-Nissimov, Yasmin Maor, Meital Elbaz, Shelly Lipman-Arens, Yonit Wiener-Well, Khetam Hussein, Efrat Orenbuch-Harroch, Regev Cohen, Oren Zimhony, Bibiana Chazan, Lior Nesher, Galia Rahav, Hiba Zayyad, Mirit Hershman-Sarafov, Miriam Weinberger, Ronza Najjar-Debbiny, Michal Chowers

**Affiliations:** 1 Infectious Diseases Unit, Samson Assuta Ashdod University Hospital, Ashdod, Israel; 2 Faculty of Health Sciences, Ben Gurion University of the Negev, Beer Sheba, Israel; 3 Infectious Disease Unit, Wolfson Medical Center, Holon, Israel; 4 Sackler Faculty of Medicine, Tel Aviv University, Tel Aviv, Israel; 5 Department of Infectious Diseases, Tel Aviv Sourasky Medical Center; 6 Infectious Disease and Infection Control Unit, Hillel Yaffe Medical Center, Hadera, Israel; 7 Rappaport Faculty of Medicine, Technion-Israel Institute of Technology, Haifa, Israel; 8 Shaare Zedek Medical Center, Jerusalem, Israel; 9 Faculty of Medicine, Hebrew University of Jerusalem, Jerusalem, Israel; 10 Rambam Health Care Campus, Haifa, Israel; 11 Department of Clinical Microbiology and Infectious Diseases, Hadassah Hebrew University Medical Center, Jerusalem, Israel; 12 Infectious Diseases Unit, Sanz Medical Center, Laniado Hospital, Netanya, Israel; 13 Infectious Diseases Unit, Kaplan Medical Center, Rehovot, Israel; 14 Infectious Diseases Unit, Emek Medical Center, Afula, Israel; 15 Infectious Disease Institute, Soroka Medical Center, Beer Sheba, Israel; 16 Infectious Diseases Unit, Sheba Medical Center, Tel Hashomer, Israel; 17 Infectious Disease Unit, The Baruch Padeh Medical Center, Tiberias, Israel; 18 The Azrieli Faculty of Medicine in the Galilee, Bar Ilan university, Safed, Israel; 19 Bnai Zion Medical Center, Haifa, Israel; 20 Shamir (Assaf Harofe) Medical Center, Zerifin, Israel; 21 Carmel Medical Center, Haifa, Israel; 22 Meir Medical Center, Kfar Saba, Israel

**Keywords:** COVID-19, Vaccine, breakthrough infection, Delta variant, Alpha variant

## Abstract

**Background:**

Changing patterns of vaccine breakthrough can clarify vaccine effectiveness.

**Aim:**

To compare breakthrough infections during a SARS-CoV-2 Delta wave vs unvaccinated inpatients, and an earlier Alpha wave.

**Methods:**

In an observational multicentre cohort study in Israel, hospitalised COVID-19 patients were divided into three cohorts: breakthrough infections in Comirnaty-vaccinated patients (VD; Jun–Aug 2021) and unvaccinated cases during the Delta wave (ND) and breakthrough infections during an earlier Alpha wave (VA; Jan–Apr 2021). Primary outcome was death or ventilation.

**Results:**

We included 343 VD, 162 ND and 172 VA patients. VD were more likely older (OR: 1.06; 95% CI: 1.05–1.08), men (OR: 1.6; 95% CI: 1.0–2.5) and immunosuppressed (OR: 2.5; 95% CI: 1.1–5.5) vs ND. Median time between second vaccine dose and admission was 179 days (IQR: 166–187) in VD vs 41 days (IQR: 28–57.5) in VA. VD patients were less likely to be men (OR: 0.6; 95% CI: 0.4–0.9), immunosuppressed (OR: 0.3; 95% CI: 0.2–0.5) or have congestive heart failure (OR: 0.6; 95% CI: 0.3–0.9) vs VA. The outcome was similar between all cohorts and affected by age and immunosuppression and not by vaccination, variant or time from vaccination.

**Conclusions:**

Vaccination was protective during the Delta variant wave, as suggested by older age and greater immunosuppression in vaccinated breakthrough vs unvaccinated inpatients. Nevertheless, compared with an earlier post-vaccination period, breakthrough infections 6 months post-vaccination occurred in healthier patients. Thus, waning immunity increased vulnerability during the Delta wave, which suggests boosters as a countermeasure.

## Introduction

The Israeli coronavirus disease (COVID-19) vaccination campaign, using a 3-week interval, 2-dose regimen of Comirnaty (BNT162b2 mRNA, Pfizer/BioNTech), began in December 2020; a coverage of 55% of the entire Israeli population was reached by the end of May 2021. This campaign was associated with a major impact, with SARS-CoV-2 daily incidence decreasing from above 90/100,000 inhabitants in mid-January 2021 to below 0.2/100,000 inhabitants by the end of May [1]. The immense impact of the Israeli vaccination campaign [2] led to the relaxation of most physical distancing restrictions, international travel and mandatory mask use by May 2021. 

Despite the high vaccination rate, breakthrough infections still occurred; we have previously reported a cohort of fully vaccinated hospitalised patients with breakthrough infections between January–April 2021, a time when the majority of infections were due to the severe acute respiratory syndrome coronavirus 2 (SARS-CoV-2) Alpha (Phylogenetic Assignment of Named Global Outbreak (Pango) lineage designation B.1.1.7) variant [3]. This report included 172 patients who were mostly 64 years and older, with a high prevalence of comorbidities and immunosuppression. 

Starting in June 2021, an outbreak of the SARS-CoV-2 Delta variant (Pango lineage designation B.1.617.2) emerged in Israel with a daily incidence increasing to more than 50/100,000 inhabitants by August 2021. This outbreak affected both unvaccinated and vaccinated populations, with daily incidence exceeding 60/100,000 and 40/100,000 inhabitants, respectively [1]. Two major differences between these two periods were the prevalent variant (Delta vs Alpha) [1] and the interval from vaccination, which was shown to cause waning vaccine-induced immunity [4-7].

In this study, we aimed to characterise hospitalised patients with breakthrough infections during the Delta variant wave and compare them to unvaccinated hospitalised cases during the same period. Furthermore, we sought to determine the differences between breakthrough infections during the separate Alpha and Delta variant-dominated waves.

## Methods

### Study setting and patient characteristics

A multicentre cohort study included hospitalised adult patients (aged ≥ 18 years) in 18 participating hospitals, which together comprised 75% of all COVID-19-dedicated beds for adults in Israel during the study period. 

Inclusion criteria differed between the two study phases, characterised by circulation of the SARS-CoV-2 Alpha or Delta variants. In the first phase (25 January–30 April 2021), the Alpha variant was found in 74% of sequenced SARS-CoV-2 isolates in Israel, and the Delta variant was found in 0.06% (Personal communication: A Mizrahi, Israel Ministry of Health, 15 August 2021). We included patients who received two doses of Comirnaty at least 7 days before onset of disease, had a PCR-confirmed diagnosis of SARS-CoV-2 infection, and were hospitalised in a COVID-19-dedicated unit. These patients comprised the ‘vaccinated Alpha wave’ cohort (VA). In the second phase (1 June–9 August 2021), the Delta variant was found in 97% of sequenced SARS-CoV-2 isolates in Israel, and the Alpha variant in only 2%. In this phase, patients were divided into a ‘vaccinated Delta wave’ (VD) cohort according to the aforementioned criteria of the VA cohort, and a ‘non-vaccinated Delta wave’ (ND) cohort. We excluded patients who received only one vaccine dose, were less than 1 week after the second dose, had a history of previous COVID-19, women in labour admitted to maternity wards and patients admitted during the transition period between the Alpha and Delta variants (May 2021).

### Clinical data and diagnostic testing

Clinical data were retrieved from the patients' electronic medical records by experienced infectious diseases specialists according to a predefined questionnaire and entered into a de-identified database. We recorded the condition of the patient upon admission, and the highest level of severity of COVID-19 reached during the hospital stay. Severity of illness was categorised according to the United States National Institute of Health criteria [8]; (i) asymptomatic/presymptomatic infection: positive virologic test, (ii) mild: any typical symptom of COVID-19 without dyspnoea or chest X-ray findings, (iii) moderate: clinical or radiological evidence of lower respiratory tract illness, with an oxygen saturation ≥ 94%, (iv) severe: oxygen saturation < 94% or respiratory rate > 30/minute and (v) critical: respiratory failure, septic shock or multiorgan failure. The primary outcome of the study was a composite of mechanical ventilation or in-hospital death, referred to as poor outcome.

SARS-CoV-2 PCR testing was performed using various assays at the participating centres. Quantitation cycle (Cq) values were reported according to specific gene targets but were analysed together with the lowest Cq value of any gene-target chosen as a surrogate for the viral load. Quantitative anti-spike (S) antibody tests were performed at each hospital using different validated commercial assays, but in order to harmonise the various assays, we used only the qualitative result of the test (Supplementary Materials S1: Specifications of laboratory methods used in study hospitals). 

### Statistical analysis

Variables were compared between the VD vs ND cohort, and between VD vs VA cohorts. Categorical variables were compared using Chi-squared or Fisher's exact tests, and continuous variables were compared using an independent samples t-test or a Mann–Whitney test. Several logistic regression analyses were performed. The primary analysis evaluated predictors for a poor outcome between vaccinated and unvaccinated patients during the Delta wave, and between Delta and Alpha breakthrough infections. An additional analysis evaluated the differences between the cohorts, including populations characteristics (sex, age, comorbidities) and the outcome in each cohort. Regression was done on clinically meaningful variables, and variables with p < 0.05 on univariant analysis with the enter method. All tests were two-tailed. IBM SPSS 25 was used for all analyses.

## Results

**Figure 1:**
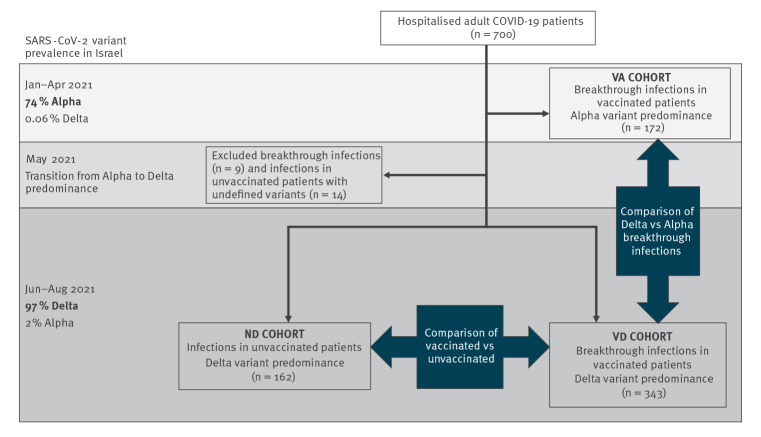
Flowchart diagram of patient inclusion and cohort selection according to period of hospital admission and COVID-19 vaccination status, Israel, January–August 2021 (n = 700)

COVID-19: coronavirus disease; SARS-CoV-2: severe acute respiratory syndrome coronavirus 2.

During the first phase of the study, 172 fully vaccinated patients were included. Within the included patients with a sequencing result, 31/36 isolates were found to be the Alpha variant, and the remaining isolates were wild-type SARS-CoV-2 (n = 3) or the Beta (n = 2) variant. This group is considered as breakthrough Alpha variant infections, referred to as VA. During the second phase of the study, 343 fully vaccinated and 162 unvaccinated patients were included. Within the included patients, 33 cases had a sequencing result reported to the study site, all of which were the Delta variant. These patients were therefore categorised as breakthrough Delta variant infections, referred to as VD, and non-vaccinated Delta variant infections, referred to as ND. Twenty-three additional patients (9 vaccinated, 14 unvaccinated) who were admitted during May were excluded, as this month had a mixed prevalence of Alpha and Delta variants. Figure 1 displays the flowchart of patients' inclusion and cohort selection. Data on outcomes were updated 11 weeks after the admission of the last patient.

### Comparison between breakthrough infections and infections in unvaccinated patients during the Delta variant wave 

Compared with unvaccinated patients (ND), the patient cohort with a breakthrough infection (VD) were 20 years older (median: 75 years (interquartile range (IQR): 67–84) vs 55 years (IQR: 42–70); p < 0.001), had a higher proportion of males (193/343 (56.3%) vs 72/162 (44.4%); p = 0.013), and resided more commonly in long-term care facilities (62/343 (18.1%) vs 4/162 (2.5%); p < 0.001) (Table 1). 

**Table 1:** Comparison between unvaccinated (ND cohort) and Comirnaty-vaccinated patients (VD cohort) hospitalised with COVID-19 during the SARS-CoV-2 Delta variant wave, Israel, June–August 2021 (n = 505)

Characteristics	ND cohort Unvaccinated (n = 162)		VD cohort Vaccinated^a^ (n = 343)		p value
	n	%	n	%	
Sex					
Male	72	44.4	193	56.3	0.013*
Female	90	55.6	150	43.7	
Age in years, median (IQR)	55	42–70	75	67–84	< 0.001*
LTCF residence	4	2.5	62	18.1	< 0.001*
Comorbidities					
None	59	36.4	38	11.1	< 0.001*
Diabetes mellitus	41	25.3	148	43.1	< 0.001*
Hypertension	63	38.9	228	66.5	< 0.001*
Obesity	31/144	21.5	67/290	23.1	0.807
Ischaemic heart disease	18	11.1	99	28.9	< 0.001*
Congestive heart failure	9	5.6	53	15.5	0.002*
Chronic renal failure	11	6.8	71	20.7	< 0.001*
Chronic lung disease	19	11.7	63	18.4	0.070
Chronic liver disease	3	1.9	8	2.3	0.764
Dementia	11	6.8	67	19.5	< 0.001*
Cancer	7	4.3	58	16.9	< 0.001*
Immunosuppression (any)	9	5.6	47	13.7	0.009*
SARS-CoV-2 PCR testing^b^					
Days from symptom onset to PCR, n (median (IQR))	n = 130	3 (1–3)	n = 268	1 (0–3)	< 0.001*
Lowest Cq value, mean ± SD	21.9 ± 6		20.7 ± 6.5		0.038*
Indication for hospital admission					
Severe COVID-19	101	62.3	196	57.1	0.287
Non-severe COVID-19 or other	61	37.7	147	42.9	
Maximal severity					
Asymptomatic	5	3.1	8	2.3	0.584
Mild	38	23.5	98	28.6	
Moderate	20	12.3	47	13.7	
Severe	79	48.8	143	41.7	
Critical	20	12.3	47	13.7	
Treatment					
Oxygen	104	64.2	207	60.3	0.434
Mechanical ventilation	20/162	12.3	37/343	10.8	0.821
Inotropes	12	7.4	24	7	1.000
Renal replacement therapy	4	2.5	16	4.7	0.330
Corticosteroids	107	66	218/342	63.7	0.620
Remdesivir	53	32.7	77	22.4	0.017*
Convalescent plasma/ hyperimmune globulin	6	3.7	16	4.7	0.654
Anti-IL-6	19	11.7	20/342	5.8	0.031*
Anticoagulation, prophylaxis	109	67.3	205	59.8	0.204
Anticoagulation, therapeutic	19	11.7	43	12.5	
Outcomes					
In-hospital death	19/162	11.7	63/343	18.4	0.059
Poor outcome (death or mechanical ventilation)	27/162	16.7	72/343	21.0	0.253
Length of stay in days^c^, median (IQR)	5	3–8	3	2–7	0.002*

Cq: quantitation cycle; COVID-19: coronavirus disease; IL: interleukin; IQR: interquartile range; LTCF: long-term care facility; HFNC: high-flow nasal cannula; SARS-CoV-2: severe acute respiratory syndrome coronavirus 2; SD: standard deviation.

^a^ Vaccinated patients received 2 doses of Comirnaty (BNT162b2 mRNA, BioNTech-Pfizer) 3 weeks apart.

^b^ PCR Cq values were available for 398 patients.

^c^ Only patients discharged alive.

Categorical variables between unvaccinated and vaccinated patients were compared using Chi-squared or Fisher's exact tests, and continuous variables were compared using an independent samples t-test or a Mann–Whitney test (p < 0.05). Significant values are marked with an asterisk.

VD cohort patients had a higher percentage of comorbidities (no comorbidities in 38/343 VD (11.1%) vs 59/162 ND (36.4%); p < 0.001) and had statistically significant higher percentages of diabetes mellitus, hypertension, ischaemic heart disease (IHD), congestive heart failure (CHF), chronic renal failure (CRF), dementia and cancer. VD cohort patients were more commonly immunosuppressed (47/343 (13.7%) vs 9/162 (5.6%); p = 0.009). In a multivariate analysis to determine the difference between cohorts, significant differences were found for the parameters of male sex (OR: 1.6, 95% confidence interval (CI): 1.0–2.5; p=0.04), age (OR: 1.06; 95% CI: 1.05–1.08; p < 0.01) and immunosuppression (OR: 2.5; 95% CI: 1.1–5.5; p = 0.024) (Supplementary Figure S4: Regression analysis of VD vs ND cohort).

PCR Cq values were available for 398 patients. VD patients had a Cq value 1.2 lower on average (20.7 ± 6.5 vs 21.9 ± 6, p = 0.038) vs ND, for SARS-CoV-2 PCR tests taken 2 days earlier after symptom onset (1 (IQR: 0–3) vs 3 (IQR: 1–3) days; p < 0.001, respectively). After adjusting for the timing of testing with regression analysis, the Cq value difference was not statistically significant between cohorts (OR: 0.96; 95% CI): 0.92–1.01; p = 0.107).

**Figure 2:**
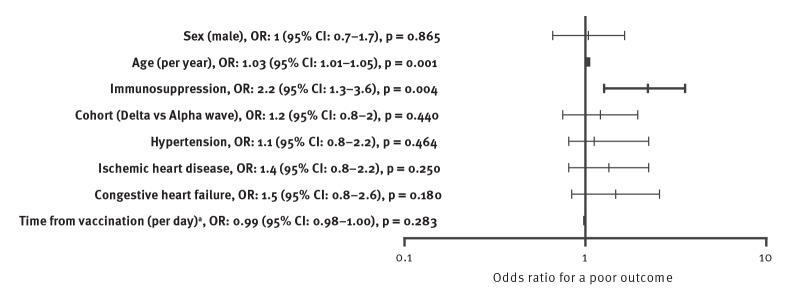
Forest plot displaying a regression analysis comparing breakthrough infections (VD cohort) to unvaccinated patients (ND cohort) during a SARS-CoV-2 Delta variant-predominant, Israel, June–August 2021 (n = 505)

CI: confidence interval; OR: odds ratio; SARS-CoV-2: severe acute respiratory syndrome coronavirus 2.

Admission indications for patients in both cohorts were similar, with 196/343 (57.1%) VD patients vs 101/162 (62.3%) ND admitted patients presenting with severe/critical COVID-19 (p = 0.287). The remaining patients were admitted with mild-to-moderate COVID-19, either as an incidental finding or because of a COVID-19-related complication. Treatment of patients in both cohorts was similar, although a lower percentage of VD patients received remdesivir and interleukin (IL)-6 antagonists. Length of stay among patients who were eventually discharged was significantly shorter for the VD cohort, with a median length of stay of 3 (IQR: 2–7) vs 5 (IQR: 3–8) days for the ND cohort (p = 0.002). The combined outcome of death or mechanical ventilation occurred in 72/343 (19.1%) of VD patients and 27/162 (16.7%) of ND patients (p = 0.460). The median age of patients with a poor outcome was 76 years (IQR: 70–86), 54.5% were male, and they had higher percentage of comorbidities including diabetes mellitus, hypertension, ischemic heart disease, chronic renal failure, dementia and immunosuppression. A univariate analysis of risk factors for a poor outcome is shown in Supplementary Table S2. In a multivariate analysis, the primary outcome was affected by age (OR: 1.04 per year; 95% CI: 1.02–1.06; p < 0.001) and immunosuppression (OR: 2; 95% CI: 1.1–3.9; p = 0.031), and not by vaccination status (Figure 2).

Vaccinated patients received 2 doses of Comirnaty (BNT162b2 mRNA, BioNTech-Pfizer) 3 weeks apart.

Vertical bars and whiskers signify OR (VD vs ND cohort) and 95% CI. Statistically significant variables are emphasised by a thicker line.

### Comparison of breakthrough infections during the Delta variant wave vs the Alpha variant wave

A detailed description of the VA cohort was reported previously [3] and is compared here to the VD cohort (Table 2).

**Table 2:** Comparison of patients hospitalised with breakthrough COVID-19 after Comirnaty vaccination in February–April 2021 (Alpha variant wave, VA cohort) and in June–August 2021 (Delta variant wave, VD cohort) Israel, January–August 2021 (n = 515)

Characteristics	VA cohort Vaccinated (Alpha wave) (n = 172)		VD cohort Vaccinated (Delta wave) (n = 343)		p value
	n	%	n	%	
Sex					
Male	121	70.3	193	56.3	0.002*
Female	51	29.7	150	43.7	
Age in years, median (IQR)	73.5	64–81	75	67–84	0.049*
Time from vaccination to admission^a^, median (IQR)	41	28–57.5	179	166–187	< 0.001*
LTCF residence	43	25	62	18.1	0.082
Comorbidities					
None	6	3.5	38	11.1	< 0.001*
Diabetes mellitus	82	47.7	148	43.1	0.348
Hypertension	124	72.1	228	66.5	0.228
Obesity	54/169	31.4	67/290	19.5	0.048*
Ischaemic heart disease	52	30.2	99	28.9	0.759
Congestive heart failure	48	27.9	53	15.5	0.001*
Chronic renal failure	56	32.6	71	20.7	0.004*
Chronic lung disease	41	23.8	63	18.4	0.163
Chronic liver disease	8	4.7	8	2.3	0.180
Dementia	32	18.6	67	19.5	0.814
Cancer	38	22.1	58	16.9	0.187
Immunosuppression (any)	64	37.2	47	13.7	< 0.001*
Pregnancy	0	0	8	2.3	0.057
SARS-CoV-2 PCR testing					
Days from symptom onset to PCR, n (median (IQR))	138	2 (0–5)	268	1 (0–3)	0.015*
Lowest Cq value, mean ± SD	23.3 ± 6.1		20.7 ± 6.5		< 0.001*
Anti-spike antibody testing					
Seropositive	52/78	66.7	93/107	86.9	0.001*
Indication for hospital admission					
Severe COVID-19	108	62.8	196	57.1	0.254
Non-severe COVID-19 or other	64	37.2	147	42.9	
Maximal severity					
Asymptomatic	14	8.1	8	2.3	0.001*
Mild	39	22.7	98	28.6	
Moderate	14	8.1	47	13.7	
Severe	66	38.4	143	41.7	
Critical	39	22.7	47	13.7	
Treatment					
Oxygen	111	64.5	207	60.3	0.387
Mechanical ventilation	23	13.4	37	10.8	0.388
Inotropes	20	11.6	24	7	0.094
Renal replacement therapy	17	9.9	16	4.7	0.034*
Corticosteroids	113	65.7	218/342	63.6	0.697
Remdesivir	40	23.3	77	22.4	0.911
Convalescent plasma / hyperimmune globulin	29/171	16.9	16	4.7	< 0.001*
Anti-IL-6	11/171	6.4	20/342	5.8	0.845
Anticoagulation (prophylaxis)	106	61.6	205	59.8	0.252
Anticoagulation (therapeutic)	28	16.3	43	12.5	
Outcomes					
In-hospital death	39	22.7	63	18.4	0.247
Poor outcome (death or mechanical ventilation)	46	26.7	72	21	0.143
Length of stay in days^b^, median (IQR)	5	2–10.3	3	2–7	0.025*

Cq: quantitation cycle; COVID-19: coronavirus disease; IL: interleukin; IQR: interquartile range; LTCF, long-term care facility; HFNC: high-flow nasal cannula; SARS-CoV-2: severe acute respiratory syndrome coronavirus 2; SD: standard deviation.

^a^ Days from second dose of Comirnaty (BNT162b2 mRNA, BioNTech-Pfizer) to hospitalisation.

^b^ Patients discharged alive only.

A detailed description of the VA cohort can be found in reference [3]. Significant values are marked with an asterisk.

The median time from vaccination to hospital admission for breakthrough infections was 179 (IQR: 166–187) vs 41 (IQR: 28–58) days for VD and VA patients, respectively (p < 0.001). Compared with VA, VD cohort patients were 1.5 years older (median: 75 vs 73.5 years; p = 0.049), had less comorbidities than VA patients (38/343 VD (11.1%) had no comorbidity vs 6/172 VA (3.5%); p < 0.001) and prevalence of CHF and CRF were lower. Prevalence of immunosuppression was lower in VD than VA (47/343 (13.7%) vs 64/172 (37.2%); p < 0.001). A multivariate analysis for the differences between the cohorts showed sex (OR: 0.6; 95% CI: 0.4–0.9), prevalence of CHF (OR: 0.6; 95% CI: 0.3–0.9) and immunosuppression (OR: 0.3; 95% CI: 0.2–0.5) to be significantly different between cohorts (Supplementary Figure S5: Regression analysis of VD vs VA cohorts).

PCR Cq values were available for 406 patients. VD patients had a 2.6 lower Cq value on average (20.7 ± 6.5 vs 23.3 ± 6.1, p < 0.001), in tests taken one day earlier after symptom onset (days (IQR): 1 (0–3) vs 2 (0–5), p = 0.015). After adjusting for the timing of testing the Cq value difference between Alpha and Delta variant cohorts was still statistically significant (OR: 0.93; 95% CI: 0.89–0.97, p = 0.001).

Serological tests for anti-S antibodies were performed for 185 patients, a median of 4 days (IQR 2–8) after disease onset. Among VD patients, 93/107 (86.9%) were seropositive vs only 52/78 (66.7%) of the VA patients (p = 0.001). Anti-S seropositivity was strongly associated with immune status, with 35/49 (71.4%) of immunosuppressed patients vs 22/115 (19.1%) of immunocompetent patients found to be seronegative for anti-S antibodies (p < 0.001).

**Figure 3:**
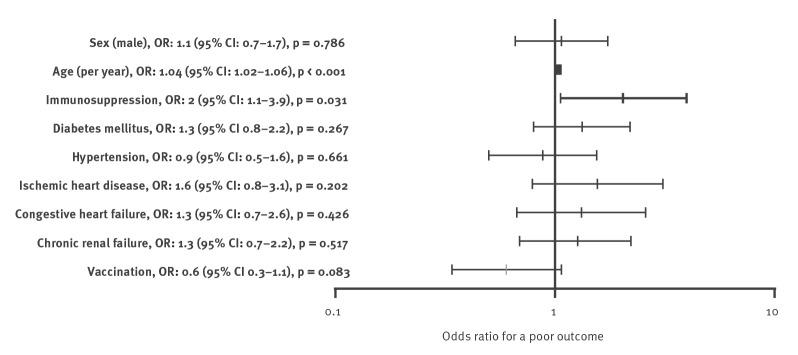
Forest plot displaying a regression analysis of breakthrough infections during the entire study period of SARS-CoV-2 Alpha variant (VA) and Delta variant (VD) waves, Israel, January–August 2021 (n = 515)

CI: confidence interval; OR: odds ratio; SARS-CoV-2: severe acute respiratory syndrome coronavirus 2.

^a^ The time from vaccination to hospital admission. Vaccinated patients received 2 doses of Comirnaty (BNT162b2 mRNA, BioNTech-Pfizer) 3 weeks apart.

Admission indications for patients in both cohorts were similar, with 196/343 (57.1%) VD patients vs 108/172 (62.8%) VA patients admitted due to severe COVID-19 (p = 0.254). The treatment of patients in the two cohorts was mostly similar. A lower percentage of VD patients received renal replacement therapy and treated with convalescent plasma therapy. Length of stay for patients that were discharged alive was significantly shorter for the VD vs the VA cohort, with a median of 3 (IQR: 2–7) vs 5 (IQR: 2–10.3) days (p = 0.025) respectively. The combined outcome of death or mechanical ventilation occurred in 72/343 (21%) of VD patients and 46/172 (26.7%) of VA patients (p = 0.143). The median age of patients with poor outcome was 77 (IQR: 71–86), 61.9% were men, they had higher percentage of comorbidities including: hypertension, ischaemic heart disease, dementia, and immunosuppression. A univariate analysis of risk factors for a poor outcome is shown in Supplementary Table S3: Univariate analysis comparing hospitalised breakthrough COVID-19 patients with a poor outcome. In a multivariate analysis, the primary outcome was affected by age (OR: 1.03 per year; 95% CI: 1.01–1.05; p = 0.001) and immunosuppression (OR: 2.2; 95% CI: 1.3–3.6, p = 0.004), and not by the variant/time period (Figure 3).

Vertical bars and whiskers signify OR for a poor outcome (in-hospital death or mechanical ventilation) and 95% confidence intervals (CI). Statistically significant variables are emphasised by a thicker line.

## Discussion

In this multicentre study, we compared a cohort of fully vaccinated individuals with a breakthrough COVID-19 from a Delta variant infection (median of 25.6 (IQR: 24–27) weeks post vaccination) to two distinct cohorts: one cohort of unvaccinated COVID-19 patients with Delta variant infections, and the second cohort of fully vaccinated patients with breakthrough COVID-19 from Alpha variant infections (median of 6 (IQR: 4–8) weeks post vaccination).

The comparison of vaccinated patients with breakthrough infections leading to hospitalisation, referred to as severe breakthrough infections, to hospitalised unvaccinated patients during the Delta wave showed a prominent difference. Patients hospitalised with breakthrough infections were 20 years older than unvaccinated patients and had a higher prevalence of immunosuppression. We assessed independent variables for poor outcome of in-hospital death or mechanical ventilation; age and immunosuppression were highly corelated with poor outcome, but vaccination was not. It is important to note though, that after adjustment for age and other differences, vaccinated patients had a trend towards a lower chance for the primary outcome of in-hospital death or mechanical ventilation, with an OR of 0.6 (95% CI: 0.3–1.1, p = 0.083). The age difference suggests that vaccination successfully prevented hospitalisation of young adults, but once hospitalised, vaccinated individuals had similar risk for poor outcome as hospitalised unvaccinated adults. Nevertheless, vaccinated patients had a 2-day shorter length of stay compared with unvaccinated ones, which translate to a lower burden on hospitals.

Severe breakthrough infections differed between the two waves. Although the primary outcome did not differ between these cohorts, there were significant differences in the patients' characteristics. During the earlier Alpha wave, more than a third of severe breakthrough infection patients were immunosuppressed, and the majority (96.5%) had comorbidities associated with COVID-19, notably obesity, diabetes mellitus, hypertension, IHD, CHF and CRF. In contrast, patients in the later Delta wave had a lower rate of immunosuppression (13.7%) and comorbidities (88.9%). Differences remained significant in the multivariate analysis, with Delta wave patients having less immunosuppression and CHF. In other words, less comorbid individuals developed severe breakthrough infection during the Delta wave. Two explanations were plausible: changes in the pathogen (different variant) and/or in the host (susceptibility). As more literature accumulates, and data from the vaccine booster initiative in Israel are available, we are inclined to favour a larger role of the later.

A lower effectiveness of Comirnaty vaccination against the Delta variant was reported in the United Kingdom (UK) shortly after the beginning of the Delta wave (May–June 2021. Decreased vaccine effectiveness (VE) of 79% for Delta vs 92% for Alpha was shown in one report [9] and 88% vs 93.7% in another [10] for prevention of SARS-CoV-2 infection, but no difference was observed in VE for prevention of hospitalisation in the UK [9,11] or the United States [12,13]. These differences matched results of neutralising assays using sera from vaccinated individuals, showing decreased neutralising titres [14,15]. Further studies showed that waning immunity can significantly decrease VE, as early as 4 months after the primary vaccination series [5-7,16-19]. For example, two nationwide analyses from Israel showed lower protection from infection and from severe disease with the Delta variant with increasing time from vaccination [5,6]. Again, these findings correlated with anti-spike antibody waning, with titres reported to wane ca twofold within 70 days [4]. Lastly, the most significant proof for waning vaccine protection comes from the result of the national campaign for booster vaccination in Israel, with effectiveness increasing more than 11-fold for symptomatic infection and 19-fold for severe infection, beginning a week post-booster [20]. The results of our study support these data on waning immunity, as breakthrough infections during the Delta-wave involved patients with fewer comorbidities and immunosuppression compared with the Alpha-wave. The former patients were vaccinated a median of 25.6 weeks before admission, while the later were ‘freshly’-vaccinated, with a median time of 6 weeks before admission. The immunogenicity of COVID-19 vaccines is known to be lower in immunosuppressed patients [21-26]. During the Alpha wave, more than a third of severe breakthrough infections affected patients with known immunosuppression, and a third of these patients had no detectable anti-S antibodies even post-infection. This emphasises the limited protection that can be afforded even by an extremely effective vaccine in susceptible patients during significant community transmission. In contrast, severe breakthrough infections during the Delta wave ensued in a mostly old but an apparently immunocompetent population (86.3%), and a higher percentage of post-infection seropositive cases (86.9%), merely due to a more transmissible variant infecting a person with a waning immune response.

Evaluation of median Cq values in our cohorts showed a similar value for vaccinated and unvaccinated individuals with the Delta variant, and a lower Cq value in Delta variant breakthrough cases compared with Alpha breakthrough cases. These findings from hospitalised severe patients agree with previous reports [27-30].

Another significant difference between the three cohorts was the male sex predominance among COVID-19 cases in each of the scenarios. Beginning with the first reports out of China, male predominance was evident, especially in those with severe and critical disease [31-34]. In our cohort of VA, males comprised ca 70% of the cohort. This decreased to 56% in the VD cohort, with the difference remaining significant after adjustment (OR: 0.6; 95% CI: 0.4–0.9). In the ND cohort, a female predominance was observed, with males comprising only 44% of the cohort with an OR of 1.59 (95% CI: 1.02–2.46), of vaccinated compared with unvaccinated. We cannot provide an explanation for this trend and suggest further investigation and corroboration from other cohorts.

The strengths of this study include its multicentre design, including the majority of Israel's general hospitals and the detailed and systematic collection of individual clinical data by infectious diseases specialists. However, some limitations are to be noted. The data collection method is dependent on the accuracy of the information in the electronic records, and therefore might be partial. Only data on several pre-specified comorbidities were systematically collected, and we did not perform analysis of combinations of comorbidities. Censoring bias was greatly limited by updating patients' outcomes 11 weeks after the last patient was admitted. Data on Cq values and anti-S antibodies were not available for all included patients and therefore might not be representative of the entire cohorts. Furthermore, we analysed Cq values and anti-S titres from different laboratory platforms together, while individual methods vary in the correlation of viral loads and Cq value [35], and anti-S and neutralising titres. The generalisability of these findings to other countries might be highly dependent on the timing of the COVID-19 vaccination campaign, the circulating variants and the population's age and health status. Variant identification was available for a minority of patients. Nevertheless, national monitoring during the different periods showed a clear variant predominance, which was further corroborated for a sample of cohort patients with laboratory identification. Last, we allowed inclusion of vaccinated individuals to the VD cohort 7 days after their last dose, which is earlier than usually accepted for effectiveness studies, but only two patients were eventually included within a 7–14 day period after vaccination, and the IQR for the entire cohort was 166–187.

## Conclusions

We describe distinct groups of COVID-19 patients requiring hospitalisation. Patients with breakthrough infection following a two-dose mRNA vaccination were 2 decades older than unvaccinated patients, and had more comorbidities correlated with that age difference, although importantly, once hospitalised their outcome was similar. Breakthrough infections with the less transmissible Alpha variant that occurred shortly after vaccination were often in immunosuppressed individuals with a high prevalence of comorbidities. Conversely, breakthrough infections with the more transmissible and modestly vaccine-evasive Delta variant, occurring 6 months after vaccination, were less associated with immunosuppression and frequent comorbidities. This suggests that waning immunity in immunocompetent elderly individuals has contributed to the decreased protection for these individuals. Consequently, our findings add to previous data justifying a timely vaccine booster administration as a countermeasure to avert the surge in severe illness and hospitalisations in these patients.

## Supplementary Data

358000 Supplement
