# Supplementary material for: Hospitalised patients with breakthrough COVID-19 following vaccination during two distinct waves in Israel, January to August 2021: a multicentre comparative cohort study
Source: Euro Surveill. 2022 May 19;27(20):2101026. doi: 10.2807/1560-7917.ES.2022.27.20.2101026 (PMC9121662; doi:10.2807/1560-7917.ES.2022.27.20.2101026)

This supplementary material is hosted by *Eurosurveillance* as supporting information alongside the article, on behalf of the authors, who remain responsible for the accuracy and appropriateness of the content. The same standards for ethics, copyright, attributions and permissions as for the article apply. Supplements are not edited by *Eurosurveillance* and the journal is not responsible for the maintenance of any links or email addresses provided therein.

Supplementary material:

Hospitalized patients with breakthrough COVID-19 six month following Comirnaty vaccination compared to unvaccinated inpatients and to inpatients with breakthrough infection six weeks following vaccination - a multicenter comparative cohort study

Contents:

	Page
S1: Specifications of laboratory methods used in study hospitals (PCR, serology)	2
Table S2: Univariate analysis comparing hospitalized COVID-19 patients with a poor outcome and a favorable outcome, vaccinated and unvaccinated patients during the Delta-wave.	3
Table S3: Univariate analysis comparing hospitalized breakthrough COVID-19 patients with a poor outcome (in-hospital death or mechanical ventilation) and a favorable outcome, vaccinated patients during the Alpha and Delta waves.	4
Figure S4: Forest plot displaying a regression analysis comparing breakthrough infections during a delta-predominant late wave (VD cohort) to non-vaccinated patients in the same wave (ND cohort).	5
Figure S5: Forest plot displaying a regression analysis comparing breakthrough infections during a delta-predominant late wave (VD cohort) to an alpha-predominant earlier wave (VA cohort).	6

This supplementary material is hosted by *Eurosurveillance* as supporting information alongside the article, on behalf of the authors, who remain responsible for the accuracy and appropriateness of the content. The same standards for ethics, copyright, attributions and permissions as for the article apply. Supplements are not edited by *Eurosurveillance* and the journal is not responsible for the maintenance of any links or email addresses provided therein.

S1: Specifications of laboratory methods used in study hospitals

PCR tests for SARS-CoV-2 RNA, that were used in various participating hospitals:

1. Xpert® Xpress SARS-CoV-2, Cepheid (Sunnyvale, CA, USA).
2. Simplexa™ COVID-19 direct kit, DiaSorin Molecular (Cyprus, CA, USA)
3. Allplex™ 2019-nCoV assay, Seegene (Seoul, South Korea)
4. BD SARS-CoV-2, BD Max™, BD Diagnostics (Baltimore, Ma, USA)
5. TaqPath COVID-19 RT PCR kit, ThermoFisher (Waltham, MA, USA)
6. COBAS® SARS-CoV-2 test, Roche (Rotkreuz, Switzerland)
7. Artus SARS-CoV-2 Prep&Amp UM Kit, Qiagen (Germantown, MD, USA)

For each test, cycle threshold (Ct) values for each gene target were reported, and the lowest Ct of all targets was chosen as representative of the test. As the different assays test for different gene targets and have distinct performances, Ct values should be considered only as correlates of the viral loads.

SARS-CoV-2 antibody tests used in various participating hospitals:

1. LIAISON® SARS-CoV-2 S1/S2 IgG, DiaSorin (Saluggia, Italy)
2. SARS-CoV-2 IgG, Abbott (Lake Forest, Il, USA)
3. Access SARS-CoV-2 IgG antibody test, Beckman Coulter (Nyon, Switzerland)
4. ADVIA Centaur XP/XPT SARS-CoV-2 IgG, Siemens (Malvern, PA, USA)

All the above tests are quantitative tests for SARS-CoV-2 IgG. As different hospitals used different commercial assays, and as each of these assays has an arbitrary scale of IgG levels, we have only used the qualitative result of the test (positive/negative) as per the manufacturer instructions.

This supplementary material is hosted by *Eurosurveillance* as supporting information alongside the article, on behalf of the authors, who remain responsible for the accuracy and appropriateness of the content. The same standards for ethics, copyright, attributions and permissions as for the article apply. Supplements are not edited by *Eurosurveillance* and the journal is not responsible for the maintenance of any links or email addresses provided therein.

Table S2: Univariate analysis comparing hospitalized COVID-19 patients with a poor outcome (in-hospital death or mechanical ventilation) and a favorable outcome. Included are patients admitted between June and August 2021 (delta-variant wave).

		Favorable outcome	%	Poor outcome		P value
N		406		99		
Male gender		211	52%	54	54.5%	0.646
Age, years, median (IQR)		69.8 (55-80)		76 (70-86)		**<0.001***
Vaccinated		271	66.7%	72	72.7%	0.253
LTCF residence		43	10.6%	23	23.2%	**0.001***
Comorbidity	None	88	23.9%	9	10.9%	**0.004***
	Diabetes mellitus	140	34.5%	49	49.5%	**0.006***
	Hypertension	222	54.7%	69	69.7%	**0.022***
	Obesity	78	22.2%	20	24.1%	0.713
	Ischemic heart disease	81	20%	36	36.4%	**0.001***
	Congestive heart failure	42	10.3%	20	20.2%	**0.005***
	Chronic renal failure	57	14%	25	25.3%	**0.007***
	Chronic lung disease	64	15.8%	18	18.2%	0.559
	Chronic liver disease	8	2%	3	3%	0.517
	Dementia	51	12.6%	27	27.3%	**<0.001***
	Cancer	45	11.1%	20	20.2%	**0.015***
Immunosuppression	Any	39	9.6%	17	17.2%	**0.032***

Abbreviations: IQR, interquartile range; LTCF, long-term care facility. Significant values are marked with an asterisk.

This supplementary material is hosted by *Eurosurveillance* as supporting information alongside the article, on behalf of the authors, who remain responsible for the accuracy and appropriateness of the content. The same standards for ethics, copyright, attributions and permissions as for the article apply. Supplements are not edited by *Eurosurveillance* and the journal is not responsible for the maintenance of any links or email addresses provided therein.

Table S3: Univariate analysis comparing hospitalized breakthrough COVID-19 patients with a poor outcome (in-hospital death or mechanical ventilation) and a favorable outcome. Included are fully-vaccinated patients with breakthrough infection during both alpha and delta variant waves.

		Favorable outcome	%	Poor outcome	%	P value
N		397		118		
Male gender		241	60.7%	73	61.9%	0.821
Age, years, median (IQR)		73 (64-82)		77 (71-86)		**<0.001***
Time from vaccination to admission[1] - median (IQR)		154 (48-177)		93 (39-169)		**0.026**
LTCF residence		70	17.6%	35	29.7%	**0.004***
Comorbidity	None	43	10.8%	1	0.8%	**0.001***
	Diabetes mellitus	172	43.3%	58	49.2%	0.264
	Hypertension	261	65.7%	91	77.1%	**0.020***
	Obesity	89	25.2%	32	30.2%	0.308
	Ischemic heart disease	104	26.2%	47	39.8%	**0.004***
	Congestive heart failure	66	16.6%	35	29.7%	**0.002***
	Chronic renal failure	94	23.7%	33	28%	0.343
	Chronic lung disease	76	19.1%	28	23.7%	0.276
	Chronic liver disease	12	3%	4	3.4%	0.840
	Dementia	66	16.6%	33	28%	**0.006***
	Cancer	65	16.4%	31	26.3%	**0.015***
Immunosuppression	Any	76	19.1%	35	29.7%	**0.015***
Delta variant		271	63.8%	72	61%	0.143

Abbreviations: IQR, interquartile range; LTCF, long-term care facility.

Significant values are marked with an asterisk.

This supplementary material is hosted by *Eurosurveillance* as supporting information alongside the article, on behalf of the authors, who remain responsible for the accuracy and appropriateness of the content. The same standards for ethics, copyright, attributions and permissions as for the article apply. Supplements are not edited by *Eurosurveillance* and the journal is not responsible for the maintenance of any links or email addresses provided therein.

Figure S4: Forest plot displaying a regression analysis comparing breakthrough infections during a delta-predominant late wave (VD cohort) to non-vaccinated patients in the same wave (ND cohort). Vertical bars and whiskers signify odds ratio (VD vs. ND cohort) and 95% confidence intervals (CI). Statistically significant variables are emphasized by a thicker line.

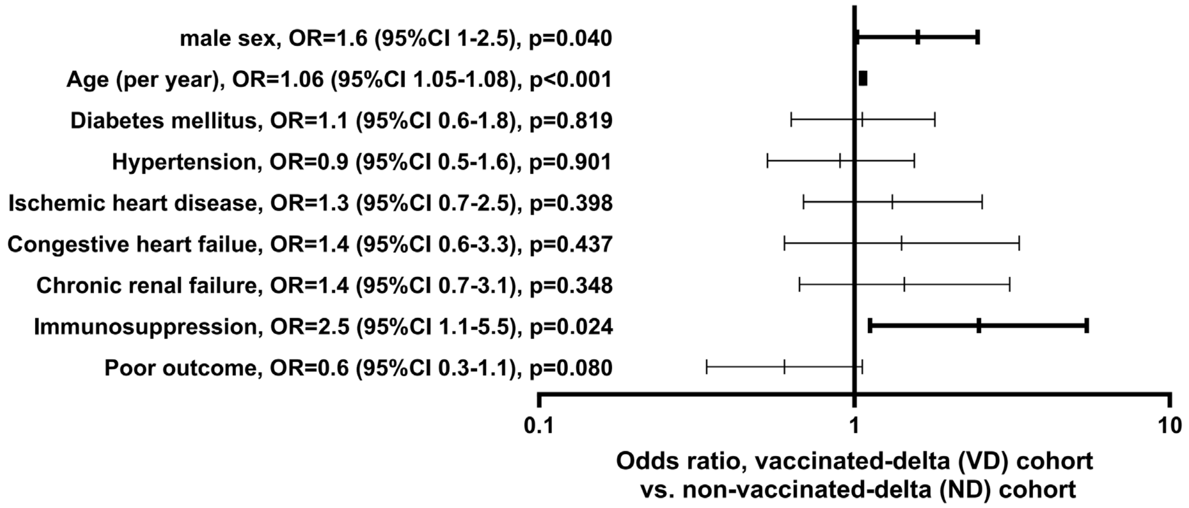

This supplementary material is hosted by *Eurosurveillance* as supporting information alongside the article, on behalf of the authors, who remain responsible for the accuracy and appropriateness of the content. The same standards for ethics, copyright, attributions and permissions as for the article apply. Supplements are not edited by *Eurosurveillance* and the journal is not responsible for the maintenance of any links or email addresses provided therein.

Figure S5: Forest plot displaying a regression analysis comparing breakthrough infections during a delta-predominant late wave (VD cohort) to an alpha-predominant earlier wave (VA cohort). Vertical bars and whiskers signify odds ratio (VD vs. VA cohort) and 95% confidence intervals (CI). Statistically significant variables are emphasized by a thicker line.

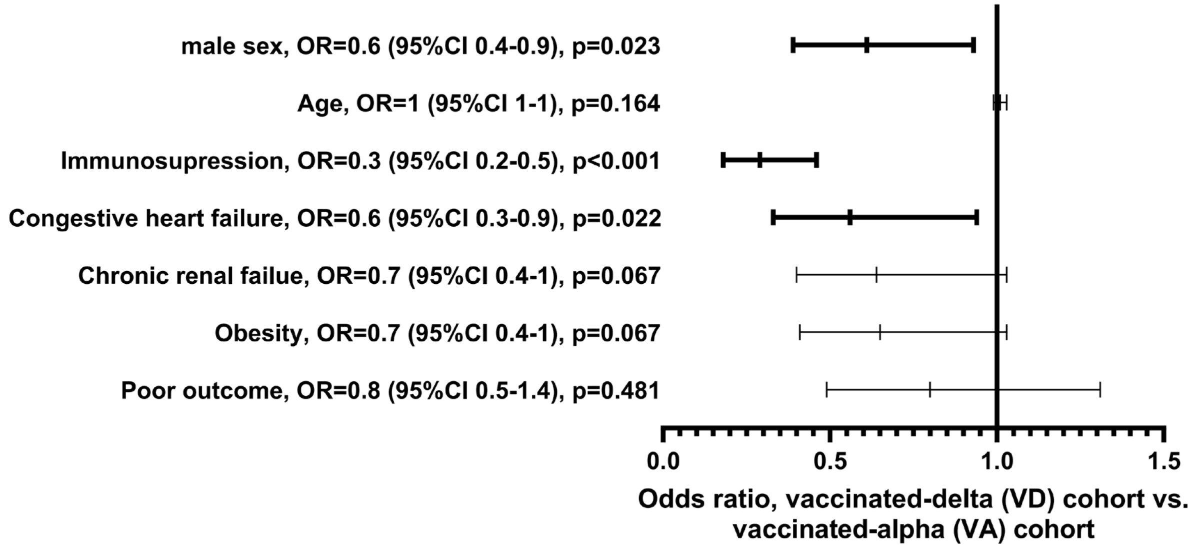

Supplement: Supplement [file 21-01026_BROSH-NASSIMOV_SUPPLEMENT.pdf]
